# Real-world effectiveness of omalizumab for severe allergic asthma treatment in Colombia

**DOI:** 10.1186/s12890-022-02246-x

**Published:** 2022-11-28

**Authors:** Carlos A. Torres-Duque, Jaime Ocampo-Gómez, Mauricio Morales Castillo, Diana Cano-Rosales, Ángela Giraldo-Montoya, Freddy Rodríguez, Isabel Palacios-Ortega, Mauricio Durán-Silva, Humberto Reynales, Elizabeth García, REXACOL Consortium, Juliana Proaños-Jurado, Alejandro Carreño, Ana M. Celis, Edgardo Chapman, Maria B. García, Mauricio González-García, Libardo Jiménez-Maldonado, Julian Londoño, Edison Morales, Mauricio Morales-Castillo, Viviana Valencia, Ingrid Vanegas, Leslie Vargas-Ramírez

**Affiliations:** 1 Present Address: Research Department, Fundación Neumológica Colombiana, Carrera 13 B No. 161 – 85, Bogotá, Colombia; 2 Universidad de La Sabana, Chía, Colombia; 3 Unidad Médico Quirúrgica ORL – UNIMEQ, Bogotá, Colombia; 4 Novartis de Colombia SA, Bogotá, Colombia; 5 Instituto Neumológico del Oriente, Bucaramanga, Colombia; 6 Universidad Tecnológica de Pereira, Pereira, Colombia; 7 IPS Universitaria, Medellín, Colombia; 8 Centro Médico Imbanaco, Cali, Colombia; 9 Centro de Atención e Investigación Médica – CAIMED, Chía, Colombia; 10 Universidad de Los Andes, Bogotá, Colombia; 11 Centro Unidad de Alergología, Barranquilla, Colombia; 12 Unidad Alergológica, Medellín, Colombia

**Keywords:** Effectiveness, Exacerbations, Omalizumab, Severe allergic asthma, Real world

## Abstract

**Background:**

The allergic phenotype is responsible for more than 50% of severe asthma cases. In a stepwise approach, add-on treatments such as anti-IgE are used for severe allergic asthma (SAA). This study was aimed to describe the real-world effectiveness of omalizumab in adult and pediatric patients with SAA in Colombia.

**Methods:**

This was an observational, non-interventional, retrospective study. Data from patients with SAA that received at least one month of treatment with omalizumab was obtained from medical records at eight sites in Colombia. Time-zero (t − 0) was defined as the date of initiation of omalizumab, and data was gathered for a 12-month period before t − 0 and a 12-month period after t − 0. Clinical outcomes, including exacerbations, were assessed at 6 and 12 months. Effectiveness of omalizumab was evaluated in terms of the reduction of the risk of exacerbations (annualized rate).

**Results:**

We included 143 patients with SAA. There was a decrease of 72.4% of the annualized rate of clinically significant asthma exacerbations during the year after omalizumab (from 1.74 before to 0.48 after) with a substantial reduction of the risk of exacerbations by 56.7% (RR [95% CI] 0.43 [0.30–0.63] *p* < 0,001).

**Conclusion:**

The use of omalizumab in Colombia as a treatment for SAA notably reduced the risk of clinically significant exacerbations. This study is the first to evaluate omalizumab real-life effectiveness in pediatric and adult patients in the country.

## Background

Asthma is a frequent and potentially serious chronic disease that causes major negative impact on individual and public health [1, 2]. In Colombia, the asthma prevalence has been previously estimated at 9% in adults older than 40 years [3] and 12.1% in overall population [4].

Severe asthma (SA) is defined as that which requires high doses of inhaled corticosteroids combined with at least any other controller drug, usually a long-acting beta agonist, corresponding to step 5 of the Global Initiative for Asthma (GINA), or which requires systemic corticosteroids (SC) use for > 50% of the previous year to prevent it from becoming ‘‘uncontrolled’’ or which remains uncontrolled despite this therapy [5, 6]. SA counts for approximately 5% of the overall population of patients of asthma [7, 8]. It is estimated that the allergic sensitization (allergic phenotype) is responsible for at least 50% of the cases of SA, this allergic phenotype is known as severe allergic asthma [SAA] [9, 10].

The immunoglobulin E (IgE) is the central actor of the allergic pathophysiological pathway [11, 12], and omalizumab, an anti-IgE recombinant humanized monoclonal antibody that binds to circulating IgE, inhibits the allergic response in asthma. From the pivotal studies [13, 14], the cumulative evidence has demonstrated the efficacy of omalizumab in reducing the asthma exacerbations, hospitalizations, and steroid utilization, and in improving asthma symptom scores, physician reported global evaluation of treatment effectiveness (GETE), pulmonary function and quality of life in moderate to severe allergic asthma in adults and children [15–18], including an exploratory systematic review and meta-analysis in Latin America [19].

Several real-world studies have corroborated the benefits of omalizumab found in clinical trials [20–25]. In Colombia, a tropical country with cities located a low, medium, and high altitudes, the national food and drug authority (i.e., INVIMA) has approved omalizumab as an add on therapy for the treatment of adults and children (older than 6 years) affected with moderate or severe persistent allergic asthma, whose symptoms cannot be adequately controlled with high dose of inhaled corticosteroids [26, 27].

Although it has been described that allergies and allergic diseases could be different in the tropics [28], one report from our country showed that the prevalence of SAA in patients with SA was 62.1% [29] similar to that generally described. However, there is still little information about allergic asthma in tropical and developing countries. In addition, there is scarce published information about the clinical use of omalizumab in asthma in Colombia [30–32]. This study was aimed to evaluate the real-world effectiveness of omalizumab in this country.

## Methods

### Study design

This is an observational, non-interventional, multi-center, retrospective, real-world study aimed to evaluate the effectiveness of omalizumab in 143 patients diagnosed with SAA, who had received omalizumab in Colombia. We used secondary data obtained from medical records at participating sites. For this purpose, a before-and-after design was used defining time-zero (t-0) as initiation of add-on therapy with omalizumab. Data was gathered for a 12-month period before t − 0 and a 12-month period after t − 0. Clinical outcomes and asthma treatment were reassessed at 6 and 12 months after t − 0, which were defined as t − 1 and t − 2, respectively. In accordance with this design, the year prior to initiation of omalizumab was used as a non-intervened control group. In this study each patient represented his own control.

### Study subjects, sample size and participant sites

We included patients over 6 years of age who had diagnosis of SAA, had received treatment for at least one month with omalizumab and had documented outcomes for a 24-month period (1 year before and one year after omalizumab).. SA was defined according to current American Thoracic Society (ATS) and European Respiratory Society (ERS) (ATS/ERS) [5] and GINA [33] consensus and SAA as additionally having a total IgE > 30 IU/mL and positive prick test or specific IgE > 0.35 kU/L. In Colombia, patients do not have a unified electronic history. In a significant number of patients, the SPT and/or IgE results were not registered in the history of the research site, but the regulatory and the Social Security System requirement for prescription of omalizumab in the country is moderate to severe allergic asthma (SAA), non-controlled with high doses of inhaled corticosteroids, an IgE > 30 up to 1500 IU/mL and/or a positive SPT. So, patients with SAA diagnosis and prescribed with omalizumab, without a registered value of IgE or SPT, as a real-world study, were also accepted for the study. The participants attended medical evaluations and received the usual clinical management for their asthma. An informed consent was signed at one participant site as requested by the institutional ethics committee (Fundación Neumológica Colombiana) and it was obtained by the site team in the first usual visit of the patients after the study approval. Informed consent was waived by the ethics committees at the other seven sites (please find the names of these ethics committees under the Declarations and Ethics approval and consent to participate section).

Exclusion criteria were defined as having other respiratory diseases different from asthma, polyposis or rhinitis, patients who had received any other biologic therapy for asthma or any investigational biologic therapy for asthma in the 24-month period or had received omalizumab as an off-label indication.

Considering previous analysis regarding real-world efficacy of omalizumab which reported reductions for asthma exacerbations and hospitalizations with Cohen’s d of 0.71 and 0.36, respectively, and selecting a size effect index > 0.25 and standard statistical thresholds of α = 0.05 and power = 0.8, a sample size of 143 subjects was calculated.

The study was carried out in eight sites from six cities in Colombia, as presented in Table 1. Data between 2013 and 2019 were collected for patients on treatment for SAA who met the selection criteria and for whom there was a 24-month period of medical history.

**Table 1:** Demographic and clinical characteristics at t0 (at starting omalizumab) (N = 143)

Characteristic	All patients (n:143)^a^
Age, median (IQR)	44 (24–57)
Sex, female, n (%)	96 (66.6)
Body Mass Index, median (IQR)	25.52 (24.36–27.47)
Type of health insurance affiliation, n (%)	
Contributory	125 (87.4)
Special regimen	12 (8.4)
Subsidized	5 (3.5)
Other	1 (0.7)
Cities and sites, n (%)	
Bogotá, 2 sites	89 (62.2)
Bucaramanga, 1 site	25 (17.5)
Pereira, 1 site	10 (7)
Medellín, 2 sites	10 (7)
Cali, 1 site	5 (3.5)
Barranquilla, 1 site	4 (2.8)
Comorbidities, n (%)	
Rhinitis	91 (64.1)
Sinusitis	27 (18.9)
Nasal polyposis	24 (16.9)
Gastroesophageal reflux	15 (10.6)
Other comorbidities	90 (63.4)
Eosinophils blood count, n (%)	
< 300 cells/μL	41 (51.9)
≥ 300 cells/μL	38 (48.1)
IgE, serum levels, n (%)	
< 30 UI/mL	2 (1.6)
≥ 30 UI/mL	118 (98.4)
Prick test result, n (%)	
Positive	71 (84.5)
Negative	13 (15.4)
Severe asthma classification criteria, n (%)	
Steps 4 or 5 or use SC in more than 50% of the previous year	116 (81.1)
High doses of IC or SC to keep asthma under control	27 (18.8)
GINA assessment for asthma control, n (%)	
Uncontrolled	76 (76)
Partly controlled	16 (16)
Well controlled	8 (8)
Deworming 12 months before t − 0, n (%)	
Yes	8 (5.6)
No	29 (20.3)
No data	106 (74.1)

^a^As it is explained in the text, not all the patients had a registered value of eosinophils, IgE and/or Prick test at starting omalizumab

### Clinical variables, measurements, and definitions

Data were obtained from the clinical records one year before and one year after the initiation of omalizumab. In addition to demographic data, we collected the following information: clinically significant exacerbations (defined as a worsening of asthma required a short cycle (three days or more) of oral corticosteroids (OCS) or, for patients who require regular long-term OCS administration, needed an increase in the OCS dose regimen (with or without hospital admission); and/or hospitalization (a length of hospital stay of 24 h or more); and/or emergency room visit (any medical attention required by a respiratory cause, including asthma)), blood eosinophils count (EOS), serum IgE level, asthma control (any tool: asthma control questionnaire [ACQ], asthma control test [ACT], [GINA] assessment), spirometry variables (forced expiratory volume in one second [FEV_1_], forced vital capacity [FVC] and FEV_1_/FVC ratio), pharmacologic treatment for asthma (inhaled and systemic corticosteroids, beta-agonists treatment, adverse reactions to omalizumab, and any other drug used).

EOS measured in the year after to omalizumab initiation was described (eosinophilic phenotype was defined as an EOS ≥ 300 cell/µL). Allergic phenotype was defined by an IgE ≥ 30 UI/mL and a positive prick test (this phenotype also includes positive serum specific IgE test, but in Colombia access to this test is limited). Current ACT, ACQ and GINA control asthma tool was used and evaluated according to the accepted cutoff.

The change of the annualized rate of clinically significant exacerbations after starting omalizumab (t − 1 and t − 2) in comparison with the previous year (t − 0) was defined as the primary endpoint**.** The description of baseline characteristics, emergency room visits, asthma-phenotype associated variables (EOS, prick test, serum total IgE and serum specific IgE), asthma-control variables (ACQ, ACT, GINA assessment), FEV_1_ and adverse events occurrence were evaluated as secondary endpoints. The correlation between the EOS (< 300 cells/μl or > 300 cells/μl) and clinical outcomes (i.e., clinically significant exacerbations, hospitalizations, and emergency room visits) was assessed as exploratory endpoint. For a simple comparison between responder and non-responder to omalizumab patients, we used a reduction of at least 50% of the number of exacerbations after one year of the use of the biologic as criteria of responder.

### Statistical analysis

Qualitative variables were expressed as absolute and relative frequencies, and quantitative variables as measurements of central tendency and dispersion, according to the Shapiro Wilk’s normality test. The annualized rate of clinically significant exacerbations was calculated for each period. To evaluate effectiveness of omalizumab, we analyzed the change of the risk of exacerbations in terms of the reduction of the annualized rate of clinically significant exacerbations. Data from patients before omalizumab initiation time (t − 0) were taken as control period (without exposure) and data after t − 1 and t − 2 were considered as intervention period (with exposure). To compare data between periods we used an analysis of variance (ANOVA) analysis. Data were analyzed for the whole group of participants and for the age groups < 18 and ≥ 18 years old. To evaluate the effectiveness of omalizumab, a multivariate analysis approach for comparing multivariate sample means was done using a multivariate analysis of variance (MANOVA) technique and taking three times (12 months before (t − 0), 6 and 12 months after starting omalizumab (t − 1 and t − 2). The number of clinically significant exacerbations was adjusted according to age, gender, and GINA classification. We performed a logistic regression analysis using improvement of control (well and partial control) as dependent variable. For the bivariate and multivariate analysis, we used age, sex, rhinitis, nasal polyposis, gastroesophageal reflux, blood eosinophils count, IgE level, SPT and asthma severity as independent variables. Statistical significance was set at *p* < 0.05, the analysis was carried out with the statistical software Stata14®.

## Results

A total of 143 patients with SAA were included in this study, with 113 (79%) of them being ≥ 18 years. Table 1 presents the demographic and clinical characteristics of the subjects; the median age was 44 years, 66.6% were female, and 64.1% had rhinitis as the most frequent comorbidity, followed by sinusitis (18.9%) and nasal polyposis (16.9%). Total EOS ≥ 300 cells/µL was observed in 48.1%, total IgE serum levels > 30 Ul/mL in 98.4% and positive prick test in 85.5%.

**Fig. 1:**
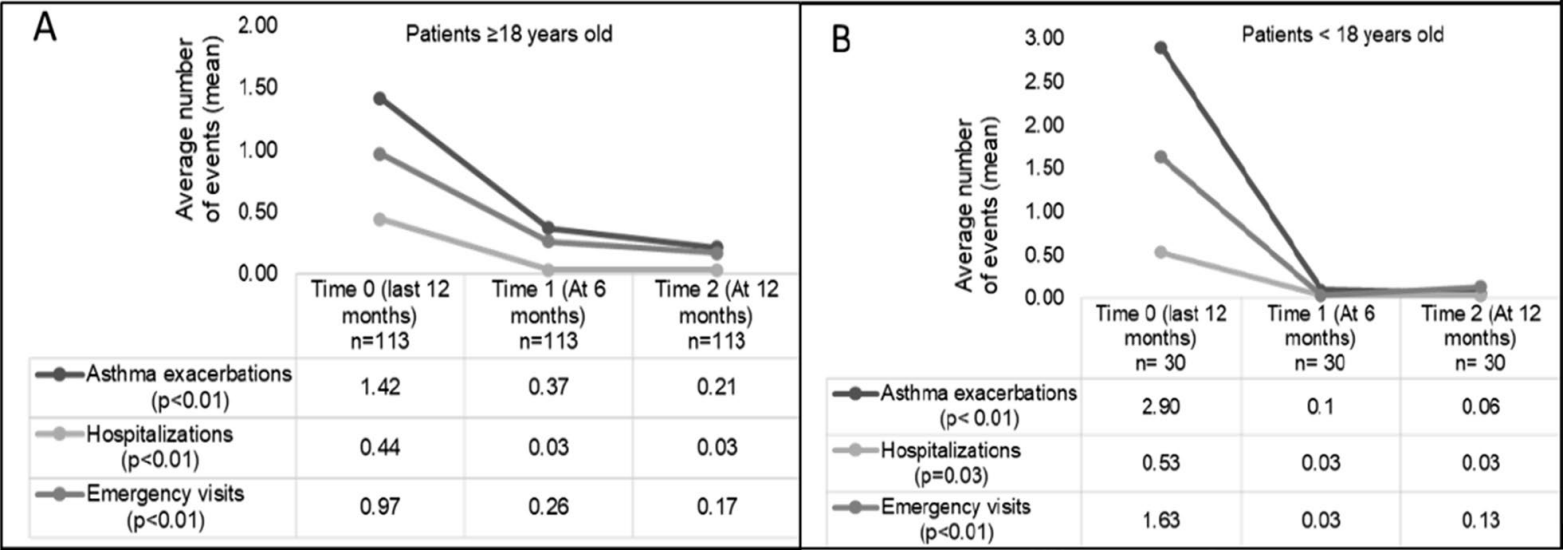
Change of number of clinically significant asthma exacerbations, hospitalizations and emergency visits after starting omalizumab (time 0), at 6 months (time 1), and at 12 months (time 2). Boxes show the annualized rate of clinically significant asthma exacerbations, hospitalizations and emergency visits. Panel A: patients ≥ 18 years old. Panel B: patients < 18 years old. A significant reduction was found in the three outcomes at times 1 and 2

The annualized rate of clinically significant asthma exacerbations was 1.74 in pre-omalizumab period and 0.48 in post-omalizumab period, with a reduction of 72.4% (Fig. 1). Omalizumab reduce the risk of exacerbations by 56.7% (RR [95% CI]: 0.43 [0.30–0.63] *p* < 0.001). The annualized rate of hospitalizations in pre-omalizumab period was 0.46 compared with 0.15 in post-omalizumab period, with a reduction of 67.4%. The annualized rate of emergency visits was 1.11 in pre-omalizumab period and 0.34 in post-omalizumab period, with a reduction of 69.4%.

**Fig. 2:**
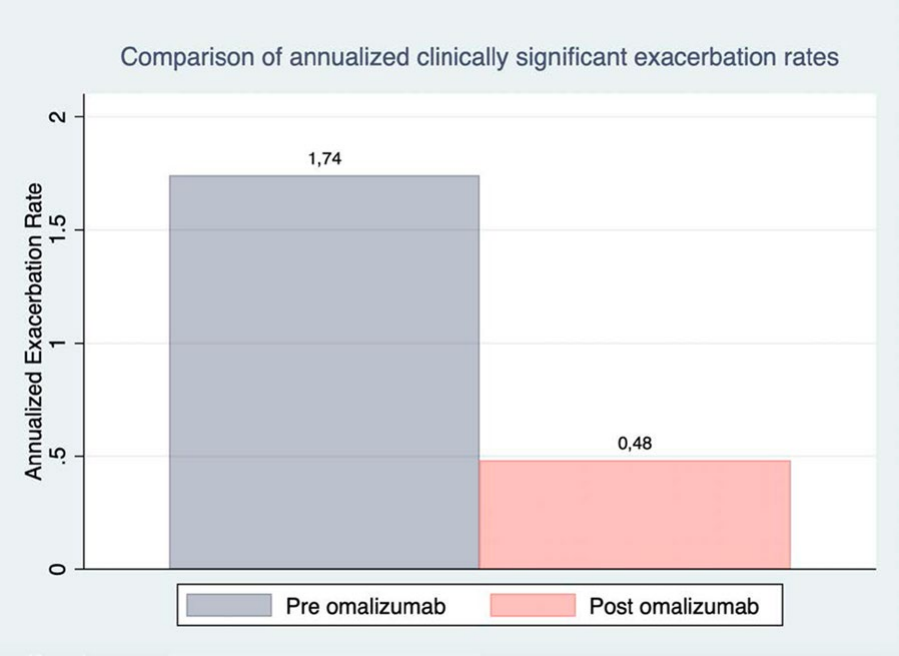
Comparison of annualized clinically significant exacerbation rates. A reduction of the annualized rate of clinically significant exacerbation was observed between the observed periods before and after Omalizumab use (n = 143)

Moreover, a significant decline of the number of exacerbations, hospitalizations and emergency visits was found at t − 1 and t − 2 both in patients < 18 and ≥ 18 years old (*p* < 0.05 for all outcomes) (Fig. 2). According to patient’s age and after starting omalizumab t − 1 and t − 2, the incidence rate of clinically significant asthma exacerbations in patients ≥ 18 years old changed from 1.42 in the pre-omalizumab period to 0.37 at t − 1 (73% reduction) and to 0.21 at t − 2 with an additional reduction of 43%. The incidence rate of hospitalizations in pre-omalizumab period was 0.44, in period t − 1 was 0,03 the reduction of the incidence rate of hospitalizations was 93% and persisted similar until t − 2. The incidence rate of emergency visits was 0.97 in pre-omalizumab period, 0.26 at t − 1 (73% reduction), and 0.17 at t − 2 (34% additional reduction) (Fig. 1). A similar pattern of change was observed in subjects < 18 years old, but with greater reductions at t-1 in this group (Fig. 2). The effect size on the reduction of exacerbations was higher in the pediatric population than the adult one (d Cohen: 1.01 [pediatric] vs. 0.33 [adults]).

**Fig. 3:**
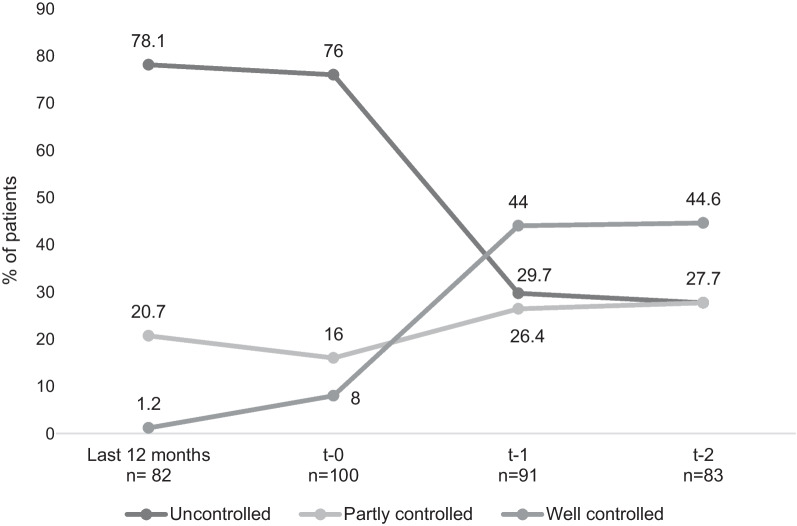
Asthma control by GINA assessment*.* Each time of measurement shows the number of patients with available information (the total n = 143). The percentage of uncontrolled patients fell considerably after omalizumab treatment starting (t − 0) with approximately 46% less patients being classified as uncontrolled at t − 1. The percentage of well controlled patients increased from 8 to 44% at t − 2

Only 57% patients had availability of data concerning asthma control using GINA assessment criteria at t-0 or at least at one timepoint (t − 1 and/or t − 2) after starting omalizumab. The percentage of uncontrolled patients decreased considerably with 46% less patients being classified as uncontrolled at t − 1 and remained similar until t − 2. On the contrary, the percentage of patients classified as well controlled increased from 8% (t − 0) to 44% (t − 2). The change in the percentage of patients initially classified as partly controlled increased with almost 14% more patients at t − 2. (Fig. 3). Not all patients were assessed consistently every 6 months; however, GINA criteria were retrospectively retrieved from medical records generated 12 months before Omalizumab use, at the time of initiation of Omalizumab and after 6 and 12 months. The number of patients whose medical records reported such data were 82, 100, 91 and 83 patients, for each moment in time respectively. Other tools of asthma control (ACT or ACQ) were significantly less used and hence this data was not reported.

**Fig. 4:**
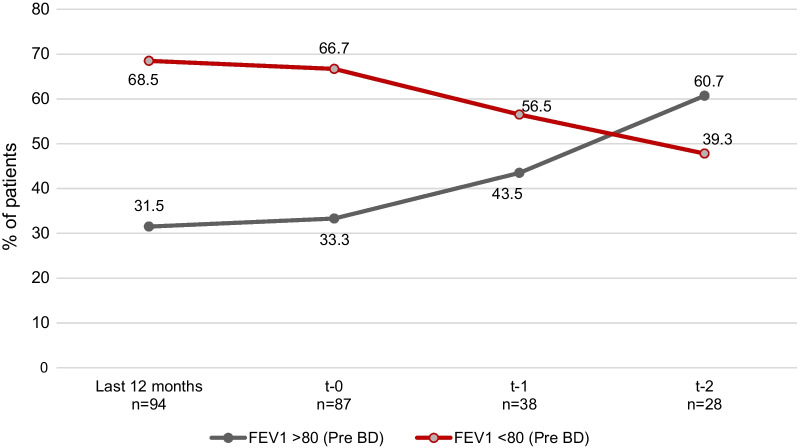
Prebonchodilator FEV_1_ assessment at starting omalizumab and follow-up. The percentage of patients with a prebronchodilator FEV_1_ < 80% consistently decreased and, on the contrary, the percentage of patients with prebronchodilator FEV_1_ ≥ 80% significantly increased after omalizumab initiation

Only 87 patients (60.8%) of the patients had the evaluation of the pre- and post-bronchodilator FEV_1_ at t − 0. Of these, 38 (43.7%) and 28 (32.2%) had a follow-up evaluation at t − 1 and t − 2 respectively. The proportion of patients with a pre-bronchodilator FEV_1_ ≥ 80% significantly increased from 31.5% at t − 0 to 43.5% and to 60.7% at t − 1 and t − 2, respectively (Fig. 4).

As per protocol inclusion criteria, at starting omalizumab (t − 0) almost all patients (96.4%) used high or medium doses of ICS combined with a long-acting beta-agonist (LABA) and 11.5% used OCS. At t − 0 some of these SAA patients also used a leukotriene modifier (80.5%) and/or a long-acting anticholinergic bronchodilator (LAMA) (32.5%). At t − 2, only 3.5% of the patients who were receiving omalizumab continued using OCS, corresponding to a reduction of 69.5% of patients requiring OCS after one year. No other significant changes in the use of other medications for asthma was observed.

As exploratory objective, we found a trend towards a higher decrease in the number of clinically significant exacerbations at both t − 1 and t − 2 in patients with EOS ≥ 300 cells/µl. However, there was no statistically significant difference, possibly because of the small sample size (n = 143 patients). We did not find significant differences between responder and non-responder to omalizumab patients, although the responder subjects tended to be younger (*p* = 0.093). Similarly, we did not find any independent variable associated with improvement of asthma control in the multivariate analysis.

Discontinuation of omalizumab was documented in 23 (16%) patients from the study population, in 6 cases (4.2%) due to adverse events as depicted in Table 2.

**Table 2:** Causes of omalizumab discontinuation at t − 1 and t − 2

Causes	t − 1 n (%)	t − 2 n (%)
Administrative barriers/health insurance company did not authorize	3 (37.5)	1 (6.67)
Adverse event	3 (37.5)	3 (20)
Worsening of symptoms/therapy change	1 (12.5)	–
Suspended by -pulmonologist decision (no explicit reason)	1 (12.5)	–
Suspended by allergologist decision (no explicit reason)	–	3 (20)
Distant home	–	2 (13.3)
Asthma exacerbation	–	1 (6.7)
Diagnosis and treatment of cancer	–	1 (6.7)
Weight gain	–	1 (6.7)
No response to treatment	–	1 (6.7)
Persistency of shortness of breath and wheezing	–	1 (6.7)
Improvement and the need of provocation tests	–	1 (6.7)

## Discussion

Our study consistently showed that in a real-world setting of patients with SAA older than 6 years in Colombia, add-on therapy with omalizumab was associated with a significant decrease of the annualized rate of clinically significant asthma exacerbations (72.4%) and a substantial reduction of the risk of exacerbations by 56.7% (RR [95% CI]: 0.43 [0.30–0.63] *p* < 0,001). Therapy with omalizumab was also associated with a significant reduction of hospitalizations and emergency room visits. Analyzed by age, these findings were similar in children and adolescents (< 18 years) and adults (≥ 18 years), although the effect size was higher in the pediatric population as it could be expected due to the greater relevance of allergy in this age group. Despite of the missing data, usual in real-world conditions, our study also showed a consistent improvement of asthma control and pulmonary function (FEV_1_), and a reduction of the proportion of patients requiring OCS.

This is the first study with a representative sample of children and adults confirming the benefits of omalizumab in patients with SAA in usual clinical practice conditions in Colombia. A previous real-life study in 61 children by Morales-Munera et al. [30] had shown similar results with a significant reduction of clinically relevant exacerbations and a decrease in controller drug consumption in 73% of patients.

The results of the current study showed superior outcomes in primary and secondary endpoints compared to other studies of similar characteristics published in both adult and children population [17, 20, 21, 23–25, 34]. We recognize that patients who are prescribed with omalizumab in our health system are closely monitored, which can improve the prescription and adherence to other pharmacological interventions and non-pharmacological measures. Although it is not possible to measure this effect in our study and it is possible that this influences the results, it is clear that the administration of omalizumab in patients with SAA in Colombia produces a benefit at least similar or greater than that described in randomized and real-life studies conducted with omalizumab.

Colombia is a tropical country with a high proportion of people living at medium and high altitudes. It is not clear what is the impact of these climate and geographic conditions on allergic sensitization and clinic expression of allergies [26], but some studies confirmed that the sensitization and allergic phenotype in asthma and severe asthma is at least as frequent (40–60%) in Colombia than that described in the literature [4, 27, 35]. Our study was not aimed to evaluate differences between the participant cities according to altitude or any other geographic, climate or environmental pollution variables.

Per protocol our patients were allergic, and they had high IgE and positive prick tests. The evaluation of EOS this population showed an almost equal distribution of eosinophilic and non-eosinophilic phenotypes which was an expected finding consistent with previously reported results [36]. Our exploratory analysis initially showed a trend towards a decrease in the mean number of clinically significant exacerbations for patients with high eosinophilic count (≥ 300 cells/μl), but it was not statistically significant due to the small subset of available data. Previous studies showed great improvement in all clinical outcomes with omalizumab regardless of EOS [22, 37, 38], which was also the case with our patients.

Our study also showed that the percentage of patients classified as uncontrolled decreased considerably with a parallel increase in the percentage of patients classified as partly controlled and well controlled. The positive impact of omalizumab on asthma control in our population is greater than that described in most of studies and similar to other studies [39, 40].

Our findings suggest a beneficial effect of omalizumab therapy on the FEV_1_. Unfortunately, we did not have enough data to trace the relevance of these findings. Some studies have reported modest benefits with greater improvements in lung function for adolescent patients after omalizumab use [41, 42].

We found a significant reduction of patients requiring OCS 12 months after omalizumab initiation. This finding has been observed in other studies with decreased corticosteroid requirements in all phenotypes after 6 months to one year of omalizumab treatment [41]. Our results suggest that complete or partial withdrawal of OCS could be considered after omalizumab initiation without compromising adequate asthma control, as supported by other studies which have reported complete discontinuation of OCS in almost 50% or more of patients after one year of omalizumab use [16, 22, 43]. The effects of long-term use of OCS for severe asthma management in adults have already been documented including acute and chronic conditions involving cardiovascular, metabolic, psychiatric and ocular diseases [24, 44]. Omalizumab may be introduced as a reasonable alternative for cases of severe allergic asthma to reduce side effects and a negative impact on quality of life.

There was not a significant reduction in the doses of the inhaled corticosteroids after 1 year of treatment with omalizumab. The participant centers are heterogeneous and usually there is not a structured plan for reduction of inhaled steroids after biologic initiation according to control improvement and exacerbations decrease. Because of the starting diagnosis was severe asthma in most of the patients and 55.4% persisted uncontrolled or partially controlled after 1 year, it is possible that treating physicians did not reduce the dose of inhaled corticosteroids in many of them and statistically significant reduction were not reached.

On the other hand, healthcare costs for patients with severe asthma may be three of four times higher than those with less complicated asthma. One study conducted in Brazil led to the conclusion that omalizumab add-on therapy could be cost-effective but nor directly, and could support the benefit of the long-term reduction in the number of exacerbations with omalizumab use [45]. Omalizumab’s clinical benefits and pharmacoeconomic features are maximized when patients are correctly selected for its use [31].

The main strength of our study is its real-life character and its representative sample which support the benefit of using of the intervention (omalizumab) in the clinical practice. In Colombia, omalizumab is available for most of the general population because its cost is covered by the National Insurance Health System. So, the starting and adherence to omalizumab are not influenced by the cost, and the subjects included in our study are representative of patients with severe allergic asthma using omalizumab in the country. We have important limitations related to the significant number of missing data and the variable quality of data acquisition which are recorded during the medical practice routine and depend on the attending physician or the medical history format (paper or electronic). Results from this study should be applied carefully to the general population in Colombia due to missing data for some of the executed analysis.

## Conclusions

In a real-life setting in Colombia, the use of omalizumab as a treatment for severe allergic asthma significantly reduces the frequency of asthma exacerbations, hospitalizations and emergency room visits due to respiratory causes, and improves the asthma control in both adult and pediatric patients. Our results support that reduction or withdrawal of OCS could be considered after omalizumab initiation. This study revealed the effectiveness of omalizumab in patients with different phenotypical characteristics with a positive response to treatment. Future research is needed in Colombia to further explore a possible relationship between phenotypic characteristics of severe allergic asthma and cost-effectiveness of omalizumab for our healthcare system.

## Data Availability

The datasets used and/or analyzed during the current study are available from the corresponding author on reasonable request.

## Abbreviations

SAA: Severe allergic asthma
SA: Severe asthma
GINA: Global Initiative for Asthma
SC: Systemic corticosteroids
IgE: Immunoglobulin E
GETE: Global evaluation of treatment effectiveness
INVIMA: Instituto Nacional de Vigilancia de Medicamentos y Alimentos, national food and drug authority in Colombia
ATS: American Thoracic Society
ERS: European Respiratory Society
OCS: Oral corticosteroids
EOS: Blood eosinophils count
ACQ: Asthma Control Questionnaire
ACT: Asthma control test
FEV1: Forced expiratory volume in 1 second
FVC: Forced vital capacity
LABA: Long-acting beta-agonist
